# A Probability Approach to the Study on Uncertainty Effects on Gamma Index Evaluations in Radiation Therapy

**DOI:** 10.1155/2011/861869

**Published:** 2011-02-07

**Authors:** Francisco Cutanda Henríquez, Silvia Vargas Castrillón

**Affiliations:** ^1^Servicio de Medicina Nuclear, Hospital General Universitario Gregorio Marañón, Calle Doctor Esquerdo, 46, 28007 Madrid, Spain; ^2^Laboratorio de Metrología de Radiaciones Ionizantes, Centro de Investigaciones Energéticas, Medioambientales y Tecnológicas, Avenida Complutense, 22, 28040 Madrid, Spain

## Abstract

Two datasets of points of known spatial positions and an associated absorbed dose value are often compared for quality assurance purposes in External Beam Radiation Therapy (EBRT). Some problems usually arise regarding the pass fail criterion to accept both datasets as close enough for practical purposes. Instances of this kind of comparisons are fluence or dose checks for intensity modulated radiation therapy, modelling of a treatment unit in a treatment planning system, and so forth. The gamma index is a figure of merit that can be obtained from both datasets; it is widely used, as well as other indices, as part of a comparison procedure. However, it is recognized that false negatives may take place (there are acceptable cases where a certain number of points do not pass the test) due in part to computation and experimental uncertainty. This work utilizes mathematical methods to analyse comparisons, so that uncertainty can be taken into account. Therefore, false rejections due to uncertainty do not take place and there is no need to expand tolerances to take uncertainty into account. The methods provided are based on the rules of uncertainty propagation and help obtain rigorous pass/fail criteria, based on experimental information.

## 1. Introduction

Modern radiation therapy aims at a high level of accuracy and, as a consequence, becomes more demanding regarding quality assurance checks (even patient-specific checks) and measurement and computation performance. The use of comparisons of two datasets consisting of a sample of measured or computed absorbed dose points covering the treatment field or a patient tomographic slice is frequently performed on a routine basis. Therefore, the acceptance method should be both straightforward and reliable. 

 Traditionally, treatment goals in radiation therapy were achieved by choosing several directions around the patient so that the dose from all the beams was conformed to the target volume, sparing healthy tissues. Nowadays, it is possible to improve the homogeneity of absorbed dose in the planning target volume (PTV) and reduce the absorbed dose to healthy organs using several fields of non-uniform intensity (IMRT) designed to combine in an optimised dose distribution inside the patient [1, 2]. 

The process is more complex than the one involved in conventional radiation therapy. The way the different beam orientations are combined could lead to practical problems, due to several issues: small and elongated beams are used, there are high dose gradients inside the fields, some features of the linear accelerator could have a noticeable effect, and treatment planning computation could not be accurate enough. These issues can make a particular plan unsuitable for treatment, and this is the reason why a comprehensive quality control of the technique and checks for each plan are often recommended [3, 4].

Two main types of patient specific checks have been recommended in the literature [3, 5].

(1) The first one consists on recomputing the plan substituting the representation of a suitable phantom for the patient representation and obtaining the 2D dose distribution on several planes inside the phantom. Radiographic or radiochromic film is inserted in the phantom in the same positions where the 2D doses where computed, and it is irradiated with the whole treatment. These films are scanned with an appropriate device and compared with the computed dose planes; this is a way to check the combined dose distribution. Some 3D measurement devices are also available [6–9].

(2) Irradiations are carried out for each beam, with the film or 2D detector placed perpendicular to the beam direction. The dose distributions have been previously computed with the treatment planning system, and a corresponding set of 2D computed dose distributions (or fluence maps) has to be compared with the measured ones. A check of each fluence map is obtained with this technique. 

In either case, a comparison of two datasets with a great number of points has to be performed. Similar situations arise when commissioning a treatment planning system, since computation results have to be checked against measurement results. This leads to the following problem.

 Given two arrays of values (absorbed dose), maybe with different spacing, find a convenient criterion to decide whether or not they can be considered as coincident for practical purposes. The dose distributions to be expected in radiation therapy can have sharp gradients in the field boundary, and possibly also inside the field, where the dose is not homogeneous. Wherever a sharp gradient is present, the result could be affected by geometrical errors (i.e., error in the position of a collimator leaf, error in the computation of the dose on the edge of the collimator leaf) and the check method should be able to cope with this. A small geometrical error is considered acceptable, but a direct comparison of the measured and reference dose in this area could result in a value out of dose tolerance. This is the reason why acceptance criteria based on distance to agreement (DTA) were developed [3–5]. DTA is the distance from the measured point to the nearest point in the reference dose distribution with the same dose. DTA tolerances are usually set for penumbra regions (field edges), and tolerances based on absorbed dose differences are used for homogeneous regions inside or outside the field. Unfortunately, there is no reasonable criterion as to whether dose difference or DTA should be used for points inside a modulated field, because there could be gradients of very different magnitude.

A solution was proposed by Low et al. [10, 11], involving the computation of a single figure of merit for the quality of the match. It has become the method of choice for acceptance of IMRT plans. It involves an artificial distance in a 3D dose space. If the dose difference tolerance is Δ*D* and the spatial tolerance is Δ*R*, then the gamma index is

(1) $$\gamma =\min\;\sqrt{\frac{D^{2}}{\Delta D^{2}}+\frac{R^{2}}{\Delta R^{2}}},$$

where *D* and *R* are the dose difference and distance to the point in the reference dataset where the square root would reach a minimum. This minimum could be an interpolated point.

A point passes the check if this index is less or equal than to 1. Δ*D* and Δ*R* are no longer strict tolerances: dose difference could be greater than Δ*D* for a point passing the gamma test; DTA could also be greater than Δ*R* for a point with a gamma less than 1; although if dose difference is greater than Δ*D* and DTA greater than Δ*R* at the same point, the gamma test fails [10]. At the same time, the absolute value of *γ* at a point where the test is not passed is a measure of the severity of the failure. 

The gamma index can be easily generalized to a 3D comparison, if DTA is computed with a 3D search [12]. A gamma filter method developed by Depuydt et al. [13] helps improve computation efficiency at the expense of not obtaining gamma values, but just checking whether or not every point is within tolerance.

It is widely acknowledged that in few occasions measured and computed datasets pass this gamma test for every measurement point, and it is customary to allow for some percentage of points failing the test [3–5, 14]. In practice, the pass rate is checked, the percentage of points in the reference dataset passes the test, and the tolerance for this rate is set according to previous experience. Therefore, the occurrence of failing points does not mean that the plan has to be rejected. This is the reason to accept a pass rate that could be less than 100%. However, there are no other grounds to accept this tolerance in pass rate, but empirical evidence, unless experimental uncertainty for the check procedure is somehow taken into account and propagated to the test indices. Basran and Woo [14] show their method to set the acceptance pass rate. They check their history of previous checks, their pass rates and whether they have been accepted or not in order to find the pass rate value corresponding to a 95% confidence. This is a purely empirical method, that ensured self-consistency, but it does not address the causes of the failing points.

Palta et al. [15] proposed a method to set tolerances in the process of commissioning, according to the observed variability. This recommendation was included in the recent report by AAPM Task Group 119 [3], to account indirectly for uncertainty in the tolerance levels. In this case, commissioning tests provide experience about variability of results that can be attributed to the experimental procedure. Analysis of the results can help set expanded tolerance levels of acceptance pass rates. 

In other kinds of comparisons (like comparisons between computed datasets), statistical information is not available and a decision about the percentage of failing points that can be tolerated has to be based on other considerations.

In this work, a novel method is presented that modifies the gamma index check, introducing uncertainty features into its computation. This method has some interesting properties: first, it is a direct propagation of experimental uncertainty, allowing for uncertainty analysis. Second, tolerance levels are not modified because of uncertainty; using this method, tolerance levels can be set to values close to the accuracy actually sought for. And third, experimental devices, computations, and their uncertainties are characterized by simple and physically meaningful parameters. Therefore, the study of the check procedure is reduced to the *a priori *study of the devices and algorithms involved.

## 2. Methods and Materials

### 2.1. Theoretical Background

Each of the datasets has been represented as several arrays of random variables. Their mean values are the values in the dataset, labelled as small-case letters with subscripts for their position and superscripts for the dataset: *x*_*ij*_^*r*^, *d*_*kl*_^*t*^,…. There is one array for each of the spatial coordinates and another for the dose. Test and reference datasets are allowed to have different values of uncertainty and array spacing, but spatial uncertainty within one of the two datasets is supposed to be isotropic. Therefore, spatial uncertainty is described by one parameter for each dataset: *σ*_*s*_^*t*^ for the test dataset and *σ*_*s*_^*r*^ for the reference dataset. Along this work, the symbol *σ* stands for standard uncertainty (one standard deviation). Similarly, *σ*_*d*_^*t*^ and *σ*_*d*_^*r*^ are dose uncertainties. Δ*R* and Δ*D* are check tolerances for the comparison between both datasets (not related to dataset uncertainty). In the next paragraphs, the computation algorithm for the comparison with uncertainty evaluation will be presented and the derivation of the algorithm can be found in the appendix.

#### 2.1.1. Probability Check for Gamma Index (2D Datasets)

A pass/fail test has to be performed for each possible pair of points, one from each dataset: point *ij* from the reference dataset and *kl* from the test dataset.

**Step 1**

Compute the following parameters:

(2) $$\begin{array}{cc} {c_{1}}^{ijkl} & =\frac{{\sigma_{d}^{t}}^{2}+{\sigma_{d}^{r}}^{2}}{\Delta D^{2}}+2\cdot \frac{{\sigma_{s}^{t}}^{2}+{\sigma_{s}^{r}}^{2}}{\Delta R^{2}}+\frac{{\left(d_{kl}^{t}-d_{ij}^{r}\right)}^{2}}{\Delta D^{2}}\\ & \;+\frac{{\left(x_{kl}^{t}-x_{ij}^{r}\right)}^{2}+{\left(y_{kl}^{t}-y_{ij}^{r}\right)}^{2}}{\Delta R^{2}}\\ & =\frac{{\sigma_{d}^{t}}^{2}+{\sigma_{d}^{r}}^{2}}{\Delta D^{2}}+2\cdot \frac{{\sigma_{s}^{t}}^{2}+{\sigma_{s}^{r}}^{2}}{\Delta R^{2}}+{\gamma_{ijkl}}^{2},\\ {c_{2}}^{ijkl} & ={\left(\frac{{\sigma_{d}^{t}}^{2}+{\sigma_{d}^{r}}^{2}}{\Delta D^{2}}\right)}^{2}+2\cdot {\left(\frac{{\sigma_{s}^{t}}^{2}+{\sigma_{s}^{r}}^{2}}{\Delta R^{2}}\right)}^{2}\\ & \;+2\cdot \left({\sigma_{d}^{t}}^{2}+{\sigma_{d}^{r}}^{2}\right)\cdot \frac{{\left(d_{kl}^{t}-d_{ij}^{r}\right)}^{2}}{\Delta D^{4}}\\ & \;+2\cdot \left({\sigma_{s}^{t}}^{2}+{\sigma_{s}^{r}}^{2}\right)\cdot \frac{{\left(x_{kl}^{t}-x_{ij}^{r}\right)}^{2}+{\left(y_{kl}^{t}-y_{ij}^{r}\right)}^{2}}{\Delta R^{4}},\\ {c_{3}}^{ijkl} & ={\left(\frac{{\sigma_{d}^{t}}^{2}+{\sigma_{d}^{r}}^{2}}{\Delta D^{2}}\right)}^{3}+2\cdot {\left(\frac{{\sigma_{s}^{t}}^{2}+{\sigma_{s}^{r}}^{2}}{\Delta R^{2}}\right)}^{3}\\ & \;+3\cdot {\left({\sigma_{d}^{t}}^{2}+{\sigma_{d}^{r}}^{2}\right)}^{2}\cdot \frac{{\left(d_{kl}^{t}-d_{ij}^{r}\right)}^{2}}{\Delta D^{6}}\\ & \;+3\cdot {\left({\sigma_{s}^{t}}^{2}+{\sigma_{s}^{r}}^{2}\right)}^{2}\cdot \frac{{\left(x_{kl}^{t}-x_{ij}^{r}\right)}^{2}+{\left(y_{kl}^{t}-y_{ij}^{r}\right)}^{2}}{\Delta R^{6}},\\ {h^{\prime }}_{ijkl} & =\frac{{{c_{2}}^{ijkl}}^{3}}{{{c_{3}}^{ijkl}}^{2}},\\ y_{ijkl} & =\left(g^{2}-\frac{{\sigma_{d}^{t}}^{2}+{\sigma_{d}^{r}}^{2}}{\Delta D^{2}}-2\cdot \frac{{\sigma_{s}^{t}}^{2}+{\sigma_{s}^{r}}^{2}}{\Delta R^{2}}-{\gamma_{ijkl}}^{2}\right)\\ & \;\cdot \frac{{c_{2}}^{ijkl}}{{c_{3}}^{ijkl}}+\frac{{{c_{2}}^{ijkl}}^{3}}{{{c_{3}}^{ijkl}}^{2}}. \end{array}$$

**Step 2**

Compute *P*_*ijkl*_ = *P*[*χ* ^2^_*h*′_*ijkl*__ > *y*_*ijkl*_] from a noncentral chi-square distribution probability function or table.

For each point in the reference dataset, the value *P*_*ij*_ = *P*[max _*kl*_(Γ_*ijkl*_) > 1] = 1 − ∏_*kl*_(1 − *P*[Γ_*ijkl*_ > 1]) is computed and the test is passed if it is less than a preset significance figure  *α*.

A global modified pass rate can be reported with the results of this test for every point in the reference dataset. A value of *α* = 0.05 is used in this study.

#### 2.1.2. Probability Distribution of Gamma (3D Datasets)

In a similar fashion, the test can be carried out for 3D datasets.

**Step 1**

Compute the following parameters:

(3) $$\begin{array}{cc} {c_{1}}^{ijklmn} & =\frac{{\sigma_{d}^{t}}^{2}+{\sigma_{d}^{r}}^{2}}{\Delta D^{2}}+3\cdot \frac{{\sigma_{s}^{t}}^{2}+{\sigma_{s}^{r}}^{2}}{\Delta R^{2}}+\frac{{\left(d_{lmn}^{t}-d_{ijk}^{r}\right)}^{2}}{\Delta D^{2}}\\ & \;+\frac{{\left(x_{lmn}^{t}-x_{ijk}^{r}\right)}^{2}+{\left(y_{lmn}^{t}-y_{ijk}^{r}\right)}^{2}+{\left(z_{lmn}^{t}-z_{ijk}^{r}\right)}^{2}}{\Delta R^{2}}\\ & =\frac{{\sigma_{d}^{t}}^{2}+{\sigma_{d}^{r}}^{2}}{\Delta D^{2}}+3\cdot \frac{{\sigma_{s}^{t}}^{2}+{\sigma_{s}^{r}}^{2}}{\Delta R^{2}}+{\gamma_{ijklmn}}^{2},\\ {c_{2}}^{ijklmn} & ={\left(\frac{{\sigma_{d}^{t}}^{2}+{\sigma_{d}^{r}}^{2}}{\Delta D^{2}}\right)}^{2}+3\cdot {\left(\frac{{\sigma_{s}^{t}}^{2}+{\sigma_{s}^{r}}^{2}}{\Delta R^{2}}\right)}^{2}\\ & \;+2\cdot \left({\sigma_{d}^{t}}^{2}+{\sigma_{d}^{r}}^{2}\right)\cdot \frac{{\left(d_{lmn}^{t}-d_{ijk}^{r}\right)}^{2}}{\Delta D^{4}}+2\cdot \left({\sigma_{s}^{t}}^{2}+{\sigma_{s}^{r}}^{2}\right)\\ & \;\cdot \frac{{\left(x_{lmn}^{t}-x_{ijk}^{r}\right)}^{2}+{\left(y_{lmn}^{t}-y_{ijk}^{r}\right)}^{2}+{\left(z_{lmn}^{t}-z_{ijk}^{r}\right)}^{2}}{\Delta R^{4}},\\ {c_{3}}^{ijklmn} & ={\left(\frac{{\sigma_{d}^{t}}^{2}+{\sigma_{d}^{r}}^{2}}{\Delta D^{2}}\right)}^{3}+3\cdot {\left(\frac{{\sigma_{s}^{t}}^{2}+{\sigma_{s}^{r}}^{2}}{\Delta R^{2}}\right)}^{3}\\ & \;+3\cdot {\left({\sigma_{d}^{t}}^{2}+{\sigma_{d}^{r}}^{2}\right)}^{2}\cdot \frac{{\left(d_{lmn}^{t}-d_{ijk}^{r}\right)}^{2}}{\Delta D^{6}}+3\cdot {\left({\sigma_{s}^{t}}^{2}+{\sigma_{s}^{r}}^{2}\right)}^{2}\\ & \;\cdot \frac{{\left(x_{lmn}^{t}-x_{ijk}^{r}\right)}^{2}+{\left(y_{lmn}^{t}-y_{ijk}^{r}\right)}^{2}+{\left(z_{lmn}^{t}-z_{ijk}^{r}\right)}^{2}}{\Delta R^{6}},\\ {h^{\prime }}_{ijklmn} & =\frac{{{c_{2}}^{ijklmn}}^{3}}{{{c_{3}}^{ijklmn}}^{2}},\\ y_{ijklmn} & =\left(g^{2}-\frac{{\sigma_{d}^{t}}^{2}+{\sigma_{d}^{r}}^{2}}{\Delta D^{2}}-3\cdot \frac{{\sigma_{s}^{t}}^{2}+{\sigma_{s}^{r}}^{2}}{\Delta R^{2}}-{\gamma_{ijklmn}}^{2}\right)\\ & \;\cdot \frac{{c_{2}}^{ijklmn}}{{c_{3}}^{ijklmn}}+\frac{{{c_{2}}^{ijklmn}}^{3}}{{{c_{3}}^{ijklmn}}^{2}}. \end{array}$$

**Step 2**

Compute *P*_*ijklmn*_ = *P*[Γ_*ijklmn*_ > 1] = *P*[*χ* ^2^_*h*′_*ijklmn*__ > *y*_*ijklmn*_] from a noncentral chi-square distribution probability function or table.

For each point in the reference dataset, the value *P*_*ijk*_ = *P*[max _*lmn*_(Γ_*ijklmn*_) > 1] = 1 − ∏_*lmn*_[1 − *P*[Γ_*ijklmn*_ > 1]] is computed and the test is passed if it is less than a preset significance figure  *α*.

As in the 2D case, a global modified pass rate can be reported with the results of this test for every point in the reference dataset. A value of *α* = 0.05 is used in this study.

### 2.2. Application

A probabilistic method to check test datasets for coincidence with a reference dataset, taking uncertainty into account, was tested with an example. It has to be remarked that for the new test to be passed, every point has to pass the test, that is, the probability test has to be passed for each pair of points drawn from the reference and test datasets. Common practice when using classic gamma test is to allow a limited percentage of points to fail the test. For the application of the present method, the probability comparison will only be passed if all points pass the test. 

**Figure 1:**
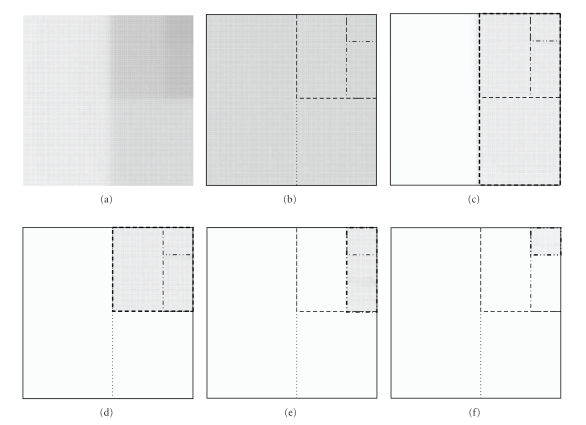
Reference dataset. (a) Composite irradiation. (b) Segment 1. (c) Segment 2. (d) Segment 3. (e) Segment 4. (f) Segment 5.

A practical example with 5 segments was set up.  Figure 1(a) shows the whole reference composite field on film and Figures 1(b), 1(c), 1(d), 1(e), and 1(f) the segments used to obtain the composite image. The composite irradiation was modified in order to introduce controlled defects.

**Case 1**

A 1 mm shift along *X* in the first segment, 1.5% more dose in the second, a 1 mm shift along *Y* in the third, and 0.5% less dose in the fourth.

**Case 2**

Same modifications, but the increment of dose in the second segment is 3% and the third is shifted 4 mm.

**Case 3**

The first segment has 2% less dose than the reference and is shifted 4 mm along *X*; the second segment has been delivered with 5% more dose; the third segment shift along *Y* is 4 mm, and the fourth has 2% less dose.

**Case 4**

All segments but the smallest one were shifted 4 mm along the *X* axis.

**Case 5**

All segments but the smallest one were shifted 4 mm along the *Y* axis.

**Figure 2:**
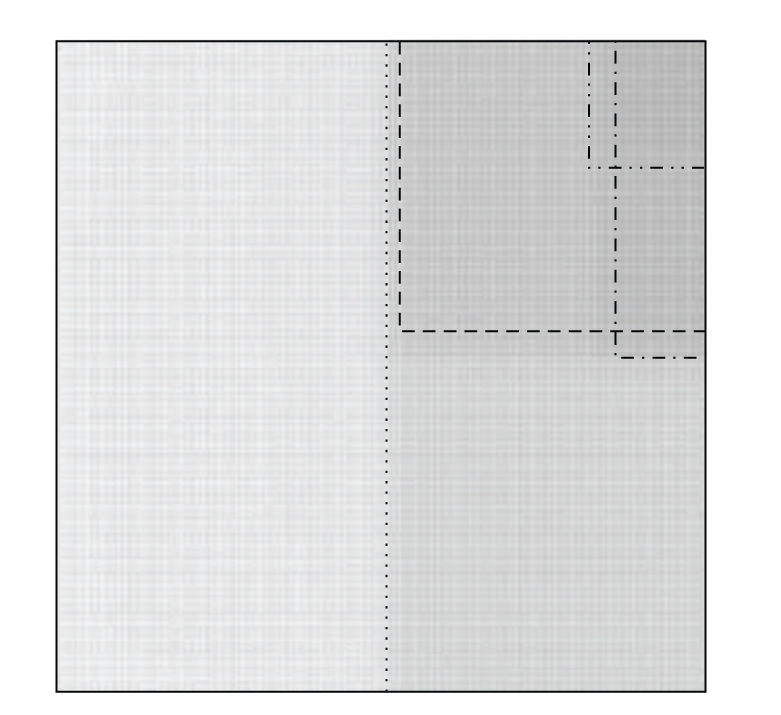
Example of setup of a test dataset by shifting and modifying segment intensity.

Therefore, each of the cases corresponds to a set of shifts and changes of intensities for every segment as exemplified in Figure 2. These controlled defects are simple enough as to make clear whether or not a test on coincidence with the reference unmodified image should pass or fail. However, the algorithm was applied with the same rigour as it would have been done for a more complex fluence pattern.

The modified planar distributions (test datasets) were compared with the original one (reference dataset) with the following uncertainty parameters: 0.2% dose and 0.5 mm, 0.5% dose and 0.5 mm, and 0.2% dose and 1 mm. Dose uncertainty is relative, and this fact has been taken into account in the computation of the indices. Tests were performed for tolerances 2% dose and 2 mm and 3% dose and 3 mm.

A function in *R* statistical software [16] was used to perform all the computations. Graphs were obtained using the “rimage” package [17].

## 3. Results

Results for the gamma test are shown in Table 1. Pass rates for a classical test are presented along with the modified test. Shaded cells contain acceptable values: 100% pass rate for the modified test and more than 98% pass rate for classical tests.

**Figure 3:**
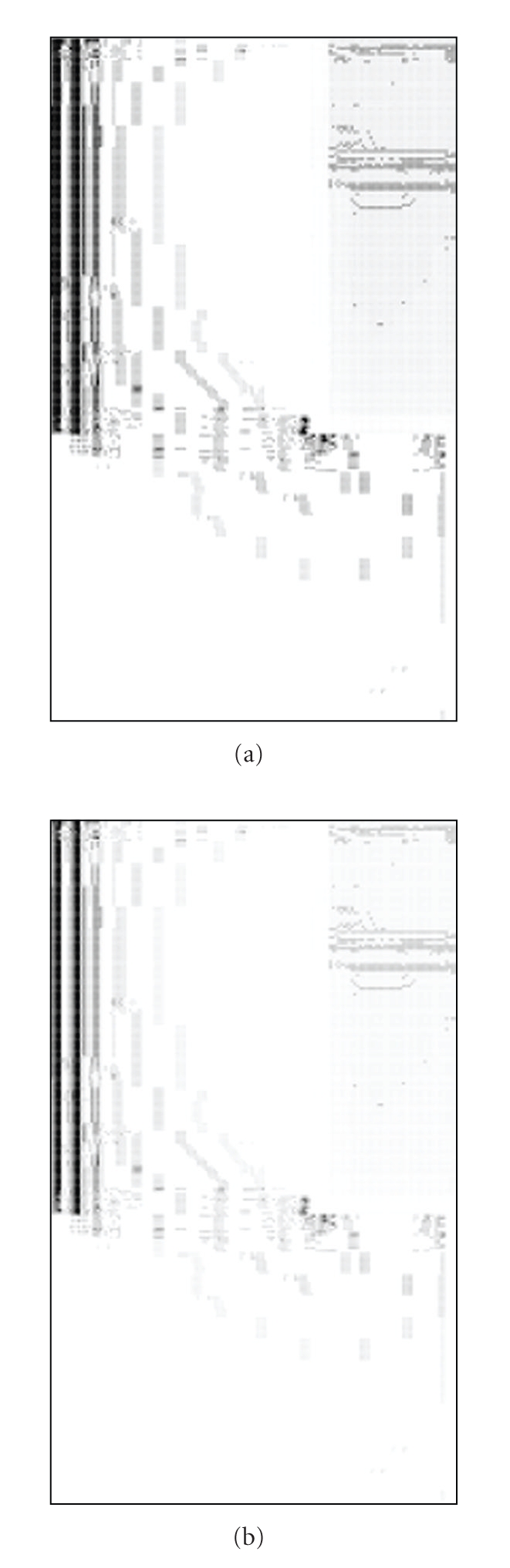
Image of pass probability for Case 2: (a) classic test with tolerances of 2 mm and 2%. (b) new test with tolerances of 2 mm and 2%.

Figure 3(a) shows a graph with points that fail the classic test for Case 2 and tolerances of 2 mm and 2%, Figure 3(b) shows the images of pass probability for the new test with 0.2% uncertainty in dose and 0.5 mm in position, the same tolerance values as in the previous case.

**Figure 4:**
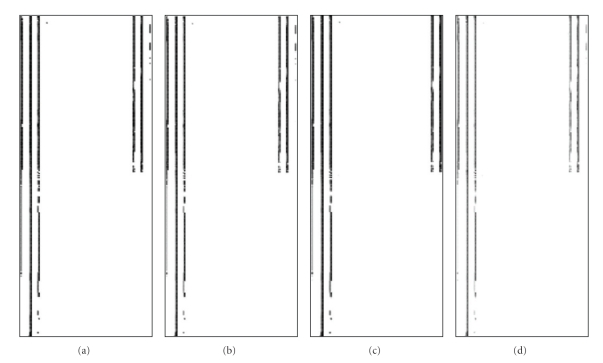
Image of pass probability for the new test for Case 4: (a) uncertainty 0.2%—0.2 mm and tolerances of 3 mm and 3%, (b) uncertainty 0.2%—0.5 mm and tolerances of 3 mm and 3%, (c) uncertainty 0.5 mm—0.5% and tolerances of 3 mm and 3%, (d) uncertainty 1.0 mm/0.2% and tolerances 3 mm and 3%.

Figure 4 shows a sequence of pass probability images for Case 4, tolerance 3 mm and 3%, and different uncertainty values: 0.2 mm/0.2%, 0.5 mm/0.2%, 0.5 mm/0.5%, and 1.0 mm/0.2%. These uncertainty values have been chosen to illustrate the method.

## 4. Discussion and Conclusions

Case 1 is *a priori *an acceptable result, Case 2 is on the limit of acceptability, and the other ones are a priori unacceptable. It is clear that the classic test failed to discard the wrong irradiations even allowing for a percentage of failing points. The usual gamma index tests would have approved every case if a 97% pass rate would be allowed and 3%—3 mm tolerances would have been used. For tolerances of 2%—2 mm, only Case 4 would have been rejected. Case 4 is an extremely undesirable plan, with an unacceptable global shift, but, interestingly enough, Case 5, with the same shift along the other axis, would have been accepted with a passing rate greater than 98%. Comparison of pass rates as well as of images in Figure 3 shows that the novel test developed in this work would have rejected cases where the standard gamma index test would allow for a great number of points in gradient areas without rejecting the comparison. Therefore, the new test is less permissive than the classic one.

On the other hand, Figure 4 and their pass rates in Table 1 show the potential misleading effect of using measurement or computing methods not suitable for the task: as uncertainty grows larger, it is possible to accept an inadequate case (Case 4), if tolerances are also too large. It can be concluded that for tolerances of 3% and 3 mm, uncertainties of 0.2% and 1 mm are enough to make the test insensitive. Tolerances of 3 mm and 3% are currently used, but these results show that they are a compromise to account indirectly for uncertainty. The test developed in this paper would work with accuracy and sensitivity with 2%—2 mm tolerances that are closer to actual physical requirements. 

The method presented in this work is potentially applicable to a broad set of comparisons: computer versus measured dose distributions for planning system commissioning, IMRT commissioning and patient checks, commissioning of measurement devices, and so forth. For any real experimental case, care should be taken to characterize its uncertainty. Furthermore, this method could be used to evaluate whether experimental uncertainties could deteriorate the sensitivity of a test. Accuracy requirements in IMRT patient plan checks are very high, and it is useful to know if the checking device uncertainty could induce the checker to accept plans too easily.

Some alternative methods have been described in the literature in order to refine the standard gamma index test; but the result is a consensus about tolerances and pass rate criteria. It is interesting to look at some conclusions in the ESTRO Booklet no. 9 [5] in the sense that it is hard to decide if test failure is related to computer system, data transfer, linear accelerator, measurement, or data analysis. A document with a similar scope is the one published by the American Association of Physicists in Medicine [3]. Both et al. [18] performed a study of check results (dose difference and distance to agreement) in order to set reasonable acceptance values for the percentage of passing points (95% for prostate, 90% for other sites) and point dose error per field (3% for prostate and 5% elsewhere). Stock et al. [19] present a strategy of primary and secondary checks. They accept checks with *γ* pass rates of 5% and prescribe further evaluation (*γ* angle, e.g.) if *γ* pass rate is greater than 5% but less than 10%. Moran [20] designed a method to allow for small range failures in the test.

In the survey performed by Nelms and Simon [21], current practice (September 2007) in the USA is presented. It is far from clear that the consensus about how to accept results from a comparison check is actually used. From these sources, it seems there is no rigorous accepted method in the literature in order to consider measurement and computation uncertainty *a priori*.

This work shows a practical application of several results about the probability distribution of quadratic forms of normal random variables. Since no *a priori* relationship between dose and position uncertainty can be assumed, the expression for the gamma index cannot be reduced to a simple noncentral chi-square random variable. This is the reason why some more refined mathematics have been used. The use of a Monte Carlo method [22] would introduce more than a million iterations for each pair of points while the three moment approximation used in this work is fast and accurate enough. Computation does not involve more iterations than a classic gamma check.

A classical test (with Δ*D* = 3%, Δ*R* = 3 mm, and a pass rate tolerance of 97%) accepts every case in this work, despite the fact that some of them were designed with controlled defects that should not be acceptable at all. Using tighter tolerances (presented, Δ*D* = 2%, Δ*R* = 2 mm), only one case is rejected (Case 4) and, oddly enough, Case 5, with the same shift as Case 4 but along the central dose edge, passes this classical test. Figure 3(b) shows that this allowance in pass rate means that points in high gradient regions are allowed to fail the test. Unless further investigation is carried out it is not clear that those failing points are due to limitations in the measurement procedure.

When the new method is used, it becomes feasible to ensure whether or not points failing a classic test are a consequence of measurement limitations. If the new test does not yield a 100% pass rate it is possible to assert that the failing points cannot have been caused solely by the measurement procedure but there is also a problem with the irradiation. Therefore, no failing points are allowed.

As pointed out previously, this novel method relates experimental features (uncertainty) with test results. A well-defined answer in terms of probability, whether or not the probability of failing a gamma test at the point *ij* is larger than  *α*, is obtained. As long as the uncertainty properties of the experimental or computational procedure have been investigated, the user is provided with a method to obtain a definite answer. On the other hand, feasibility studies become possible and it is possible to evaluate whether or not a comparison procedure uncertainty features could affect sensitivity in the test results.

## Figures and Tables

**Table 1:** Gamma results for Cases 1–5.

		Case 1	Case 2	Case 3	Case 4	Case 5
Tolerance 2%—2 mm	Dose Unc. 0.2%. Dist. Unc. 0.2 mm	**1.0000**	0.9929	0.9926	0.9696	0.9920
	Dose Unc. 0.2%. Dist. Unc. 0.5 mm	**1.0000**	0.9931	0.9930	0.9718	0.9927
	Dose Unc. 0.5%. Dist. Unc. 0.5 mm	**1.0000**	0.9932	0.9932	0.9726	0.9938
	Dose Unc. 0.2%. Dist. Unc. 1.0 mm	**1.0000**	0.9966	0.9966	0.9876	0.9972
	Classic test	**1.0000**	**0.9894**	**0.9861**	0.9615	**0.9893**

Tolerance 3%—3 mm	Dose Unc. 0.2%. Dist. Unc. 0.2 mm	**1.0000**	0.9957	0.9957	0.9847	0.9965
	Dose Unc. 0.2%. Dist. Unc. 0.5 mm	**1.0000**	0.9967	0.9968	0.9898	0.9979
	Dose Unc. 0.5%. Dist. Unc. 0.5 mm	**1.0000**	0.9968	0.9968	0.9909	0.9979
	Dose Unc. 0.2%. Dist. Unc. 1.0 mm	**1.0000**	**1.0000**	**1.0000**	**1.0000**	**1.0000**
	Classic test	**1.0000**	**0.9933**	**0.9932**	0.9794	**0.9946**

**Table 2:** 

*λ*_1_ = (*σ*_*d*_^*t*^ ^2^ + *σ*_*d*_^*r*^ ^2^)/Δ*D* ^2^	*h*_1_ = 1	*δ*_1_^2^ = (*d*_*kl*_^*t*^−*d*_*ij*_^*r*^)^2^/(*σ*_*d*_^*t*^ ^2^ + *σ*_*d*_^*r*^ ^2^)
*λ*_2_ = (*σ*_*s*_^*t*^ ^2^ + *σ*_*s*_^*r*^ ^2^)/Δ*R* ^2^	*h*_2_ = 2	*δ*_2_^2^ = ((*x*_*kl*_^*t*^−*x*_*ij*_^*r*^)^2^ + (*y*_*kl*_^*t*^−*y*_*ij*_^*r*^)^2^ + (*z*_*kl*_^*t*^−*z*_*ij*_^*r*^)^2^)/(*σ*_*s*_^*t*^ ^2^ + *σ*_*s*_^*r*^ ^2^)

## Appendices

### A. Probability Check for Gamma Index (2D Datasets)

Gamma is often described as a distance in a *N* + 1 dimensional space. If the additional dimension were a spatial one and there would be the same tolerance and uncertainty value for every spatial direction, the problem of propagating uncertainty to gamma would have been much easier. But the additional dimension, absorbed dose, has independent tolerance and uncertainty values, and the problem, in its simpler formulation, is the following one: find the probability that the sum of two normal random variables, with zero mean, different variances, and different coefficients is less than *g* ^2^.

Two sets of 2D dose distributions are defined: the reference one and the test one. Both are regular arrays but their spacing could be different. The reference points are labelled with subscripts (*ij*) and the test points with (*kl*). For each of these positions, there will be three quantities: dose *D*_*ij*_^*r*^, *x* coordinate *X*_*ij*_^*r*^, and *y* coordinate *Y*_*ij*_^*r*^. The notation for the test 2D set will be a *t* superscript instead of *r*. *X* and *Y* axes are the same for both point sets. These quantities will be considered normally distributed random variables, with mean the measured or computed values. Capital letters will refer to random variables and small letters to their means (*d*_*ij*_^*r*^, *x*_*ij*_^*r*^, *y*_*ij*_^*r*^, *d*_*kl*_^*t*^, *x*_*kl*_^*t*^, *y*_*kl*_^*t*^). Absorbed dose in the reference set has associated standard uncertainty *σ*_*d*_^*r*^ and *σ*_*d*_^*t*^ in the test set. Spatial uncertainty is isotropic in both datasets (same standard deviation for *X* and *Y*) and will be referred to with the symbols *σ*_*s*_^*r*^ and *σ*_*s*_^*t*^. Thus, we are assuming that

(A.1) $$\begin{array}{c} D_{ijkl}=D_{kl}^{t}-D_{ij}^{r}\sim N\left(d_{kl}^{t}-d_{ij}^{r},\sqrt{{\sigma_{d}^{t}}^{2}+{\sigma_{d}^{r}}^{2}}\right),\\ X_{ijkl}=X_{kl}^{t}-X_{ij}^{r}\sim N\left(x_{kl}^{t}-x_{ij}^{r},\sqrt{{\sigma_{s}^{t}}^{2}+{\sigma_{s}^{r}}^{2}}\right),\\ Y_{ijkl}=Y_{kl}^{t}-Y_{ij}^{r}\sim N\left(y_{kl}^{t}-y_{ij}^{r},\sqrt{{\sigma_{s}^{t}}^{2}+{\sigma_{s}^{r}}^{2}}\right). \end{array}$$

The squared gamma random variable Γ_*ij**kl*_^2^ = *D*_*ij**kl*_^2^/Δ*D* ^2^ + (*X*_*ij**kl*_^2^ + *Y*_*ij**kl*_^2^)/Δ*R* ^2^ is a weighted sum of normal random variables with different means and standard deviations. If this equation is written in the following way:

(A.2) $$\begin{array}{cc} {\Gamma_{ijkl}}^{2} & =\frac{{\sigma_{d}^{t}}^{2}+{\sigma_{d}^{r}}^{2}}{\Delta D^{2}}\cdot \frac{{D_{ijkl}}^{2}}{{\sigma_{d}^{t}}^{2}+{\sigma_{d}^{r}}^{2}}\\ & \;+\frac{{\sigma_{s}^{t}}^{2}+{\sigma_{s}^{r}}^{2}}{\Delta R^{2}}\cdot \left[\frac{{X_{ijkl}}^{2}}{{\sigma_{s}^{t}}^{2}+{\sigma_{s}^{r}}^{2}}+\frac{{Y_{ijkl}}^{2}}{{\sigma_{s}^{t}}^{2}+{\sigma_{s}^{r}}^{2}}\right], \end{array}$$

it becomes a weighted sum of noncentral chi-square random variables. The following random variables:

(A.3) $$\begin{array}{c} {\tilde{D}}_{ijkl}=\frac{D_{ijkl}}{\sqrt{{\sigma_{d}^{t}}^{2}+{\sigma_{d}^{r}}^{2}}}\sim N\left(\frac{d_{kl}^{t}-d_{ij}^{r}}{\sqrt{{\sigma_{d}^{t}}^{2}+{\sigma_{d}^{r}}^{2}}},1\right),\\ {\tilde{X}}_{ijkl}=\frac{X_{ijkl}}{\sqrt{{\sigma_{s}^{t}}^{2}+{\sigma_{s}^{r}}^{2}}}\sim N\left(\frac{x_{kl}^{t}-x_{ij}^{r}}{\sqrt{{\sigma_{s}^{t}}^{2}+{\sigma_{s}^{r}}^{2}}},1\right),\\ {\tilde{Y}}_{ijkl}=\frac{Y_{ijkl}}{\sqrt{{\sigma_{s}^{t}}^{2}+{\sigma_{s}^{r}}^{2}}}\sim N\left(\frac{y_{kl}^{t}-y_{ij}^{r}}{\sqrt{{\sigma_{s}^{t}}^{2}+{\sigma_{s}^{r}}^{2}}},1\right) \end{array}$$

have standard deviation 1. It is possible to use now some of the properties of the noncentral chi-squared distribution. Let *U*_*n*_ be a finite set of independent normally distributed random variables with means *μ*_*n*_ and standard deviation 1. Then, *W* = ∑_*n*_ *U*_*n*_^2^ will have a noncentral chi-square distribution *χ* ^2^(*n*, *λ*) with *n* degrees of freedom and noncentrality parameter *λ* = ∑_*n*_ *μ*_*n*_^2^. Thus,

(A.4) $$\begin{array}{cc} {\tilde{D}}_{ijkl}{\;}^{2} & =\frac{{D_{ijkl}}^{2}}{{\sigma_{d}^{t}}^{2}+{\sigma_{d}^{r}}^{2}}\sim \chi^{2}\left(1,\frac{{\left(d_{kl}^{t}-d_{ij}^{r}\right)}^{2}}{{\sigma_{d}^{t}}^{2}+{\sigma_{d}^{r}}^{2}}\right),\\ {\tilde{R}}_{ijkl}{\;}^{2} & ={\tilde{X}}_{ijkl}{\;}^{2}+{\tilde{Y}}_{ijkl}{\;}^{2}=\frac{{X_{ijkl}}^{2}}{{\sigma_{s}^{t}}^{2}+{\sigma_{s}^{r}}^{2}}+\frac{{Y_{ijkl}}^{2}}{{\sigma_{s}^{t}}^{2}+{\sigma_{s}^{r}}^{2}}\\ & \sim \chi^{2}\left(2,\frac{{\left(x_{kl}^{t}-x_{ij}^{r}\right)}^{2}+{\left(y_{kl}^{t}-y_{ij}^{r}\right)}^{2}}{{\sigma_{s}^{t}}^{2}+{\sigma_{s}^{r}}^{2}}\right). \end{array}$$

The squared gamma index is

(A.5) $${\Gamma_{ijkl}}^{2}=\frac{{\sigma_{d}^{t}}^{2}+{\sigma_{d}^{r}}^{2}}{\Delta D^{2}}\cdot {\tilde{D}}_{ijkl}{\;}^{2}+\frac{{\sigma_{s}^{t}}^{2}+{\sigma_{s}^{r}}^{2}}{\Delta R^{2}}\cdot {\tilde{R}}_{ijkl}{\;}^{2}.$$

Given a quadratic form of normally distributed variables, there always exists a transformation which reduces it to a weighted sum of noncentral chi-squared variables, corresponding to the orthogonal transformation that reduces the form to its canonical form. As a matter of fact, the previous derivation is a very simple particular case of this general result [23]. In the gamma test problem, it is necessary to evaluate the probability of the event Γ_*ij**kl*_^2^ > 1.

In the general case of a quadratic form, the noncentrality parameters are linear combinations of the means. Thus, a quadratic form of central normal variables results in a linear combination of central chi-squared variables. The normal variables involved in Γ are noncentral ones; their means are the differences between doses or between spatial coordinates in the test and the reference datasets.

Different expansions of the distribution function of a weighted sum of noncentral chi-squared variables can be found in the literature, and they could be used for this problem. Shah and Khatri [24] found a power series expansion, Ruben [25] developed series of distribution functions of central and noncentral chi-squared variables, with coefficients recursively defined, and Shah and other authors [26] proposed series involving Laguerre polynomials. There is also a simple approximation based on a study on relationships between chi-squared and Poisson variables first proposed by Patnaik [27], improved by Pearson [28], which gives accurate results especially in the tails [29]. Imhoff [23] rewrote this approximation for the weighted sum of noncentral chi-squared variables. It uses probability values for a single central chi-squared variable. This approach was used in the present work. The accuracy of this method leaves out of the question a Monte Carlo approach based on ISO recommendations [22], which would lead to a minimum of 10^7^ iterations for each pair of points.

According to Imhoff, if *Q* = ∑_*r*=1_^*m*^ *λ*_*r*_ *χ* ^2^_*h*_*r*_;*δ*_*r*_^2^_ (*δ*_*r*_^2^ being noncentrality parameters in his notation), then

(A.6) $$P\left[Q>x\right]≅P\left[{\chi^{2}}_{h\prime }>y\right],$$

where

(A.7) $$\begin{array}{c} h^{\prime }=\frac{{c_{2}}^{3}}{{c_{3}}^{2}},\\ y=\left(x-c_{1}\right)\cdot {\left(\frac{h^{\prime }}{c_{2}}\right)}^{1/2}+h^{\prime },\\ c_{j}=\overset{m}{\underset{r=1}{\sum }}{\lambda_{r}}^{j}\cdot \left(h_{r}+j{\delta_{r}}^{2}\right),\;j=1,2,3. \end{array}$$

Applying this approximation to the problem of finding *P*[Γ_*ij**kl*_^2^ > *g* ^2^], the set of parameters (2) in the main text are obtained, taking into account that the chi squared parameters are those in Table 2, taking into account that for our case, and, therefore, (2) in the main text are obtained. With those parameters *P*_*ij**kl*_ = *P*[Γ_*ij**kl*_ > *g* ^2^] = *P*[*χ* ^2^_*h*′_*ij**kl*__ > *y*_*ij**kl*_], and the probability of gamma being greater than *g* ^2^ for the points (*ij*) in the reference dataset and (*kl*) in the test dataset, taking into account spatial and dosimetry uncertainties in both datasets has been obtained.

It is possible to modify the original gamma test for the *ij* point if the probabilities

(A.8) $$P_{ij}=P\left[\underset{kl}{\max\;}\left(\Gamma_{ijkl}\right)>1\right]=1-\prod_{kl}\left(1-P\left[\Gamma_{ijkl}>1\right]\right)$$

are defined and the following criterion is set: *ij* passes the test if *P*_*ij*_ < *α* being  *α* a significance figure, set by the user as the maximum probability to be accepted.

### B. Probability Distribution of Gamma (3D Datasets)

The squared gamma random variable is now Γ_*ij**kl**m**n*_^2^ = *D*_*ij**kl**m**n*_^2^/Δ*D* ^2^ + (*X*_*ij**kl**m**n*_^2^ + *Y*_*ij**kl**m**n*_^2^ + *Z*_*ij**kl**m**n*_^2^)/Δ*R* ^2^. As long as the spatial standard uncertainties are isotropic, the same rearrangement as in the 2D case can be done

(B.1) $$\begin{array}{cc} & {\Gamma_{ijklmn}}^{2}\\ & \;\;=\frac{{\sigma_{d}^{t}}^{2}+{\sigma_{d}^{r}}^{2}}{\Delta D^{2}}\cdot \frac{{D_{ijklmn}}^{2}}{{\sigma_{d}^{t}}^{2}+{\sigma_{d}^{r}}^{2}}\\ & \;\;\;+\frac{{\sigma_{s}^{t}}^{2}+{\sigma_{s}^{r}}^{2}}{\Delta R^{2}}\cdot \left[\frac{{X_{ijklmn}}^{2}}{{\sigma_{s}^{t}}^{2}+{\sigma_{s}^{r}}^{2}}+\frac{{Y_{ijklmn}}^{2}}{{\sigma_{s}^{t}}^{2}+{\sigma_{s}^{r}}^{2}}+\frac{{Z_{ijklmn}}^{2}}{{\sigma_{s}^{t}}^{2}+{\sigma_{s}^{r}}^{2}}\right], \end{array}$$

and, defining the new random variable ${\tilde{Z}}_{ijklmn}=Z_{ijklmn}/\sqrt{{\sigma_{s}^{t}}^{2}+{\sigma_{s}^{r}}^{2}}\sim N\left(z_{lmn}^{t}-z_{ijk}^{r}/\sqrt{{\sigma_{s}^{t}}^{2}+{\sigma_{s}^{r}}^{2}},1\right)$, we obtain

(B.2) $$\begin{array}{cc} & {\tilde{D}}_{ijklmn}{\;}^{2}=\frac{{D_{ijklmn}}^{2}}{{\sigma_{d}^{t}}^{2}+{\sigma_{d}^{r}}^{2}}\sim \chi^{2}\left(1,\frac{{\left(d_{lmn}^{t}-d_{ijk}^{r}\right)}^{2}}{{\sigma_{d}^{t}}^{2}+{\sigma_{d}^{r}}^{2}}\right),\\ & {\tilde{R}}_{ijklmn}{\;}^{2}\\ & \;={\tilde{X}}_{ijklmn}{\;}^{2}+{\tilde{Y}}_{ijklmn}{\;}^{2}+{\tilde{Z}}_{ijklmn}{\;}^{2}\\ & \;=\frac{{X_{ijklmn}}^{2}}{{\sigma_{s}^{t}}^{2}+{\sigma_{s}^{r}}^{2}}+\frac{{Y_{ijklmn}}^{2}}{{\sigma_{s}^{t}}^{2}+{\sigma_{s}^{r}}^{2}}+\frac{{Z_{ijklmn}}^{2}}{{\sigma_{s}^{t}}^{2}+{\sigma_{s}^{r}}^{2}}\\ & \;\sim \chi^{2}\left(3,\frac{{\left(x_{lmn}^{t}-x_{ijk}^{r}\right)}^{2}+{\left(y_{lmn}^{t}-y_{ijk}^{r}\right)}^{2}+{\left(z_{lmn}^{t}-z_{ijk}^{r}\right)}^{2}}{{\sigma_{s}^{t}}^{2}+{\sigma_{s}^{r}}^{2}}\right),\\ & {\Gamma_{ijklmn}}^{2}=\frac{{\sigma_{d}^{t}}^{2}+{\sigma_{d}^{r}}^{2}}{\Delta D^{2}}\cdot {\tilde{D}}_{ijklmn}{\;}^{2}+\frac{{\sigma_{s}^{t}}^{2}+{\sigma_{s}^{r}}^{2}}{\Delta R^{2}}\cdot {\tilde{R}}_{ijklmn}{\;}^{2}. \end{array}$$

Using Imhoff's three moments approximation, the parameter values are the ones in equations (3) in the main text, and *P*_*ij**kl**m**n*_ = *P*[Γ_*ij**kl**m**n*_ > 1] = *P*⌊*χ* ^2^_*h*′_*ij**kl**m**n*__ > *y*_*ij**kl**m**n*_⌋. The test is *P*_*ij**k*_ < *α* with *P*_*ij**k*_ = *P*[max _*lmn*_(Γ_*ij**kl**m**n*_) > 1] = 1 − ∏_*lmn*_[1 − *P*[Γ_*ij**kl**m**n*_ > 1]].
